# Exosomes from *Plasmodium yoelii*-Infected Reticulocytes Protect Mice from Lethal Infections

**DOI:** 10.1371/journal.pone.0026588

**Published:** 2011-10-26

**Authors:** Lorena Martin-Jaular, Ernesto S. Nakayasu, Mireia Ferrer, Igor C. Almeida, Hernando A. del Portillo

**Affiliations:** 1 Poverty-Related Diseases, Barcelona Centre for International Health Research, Barcelona, Spain; 2 The Border Biomedical Research Center, University of Texas at El Paso, El Paso, Texas, United States of America; 3 Institució Catalana de Recerca i Estudis Avançats (ICREA), Barcelona, Spain; Agency for Science, Technology and Research - Singapore Immunology Network, Singapore

## Abstract

Exosomes are 30–100-nm membrane vesicles of endocytic origin that are released after the fusion of multivesicular bodies (MVBs) with the plasma membrane. While initial studies suggested that the role of exosomes was limited to the removal of proteins during the maturation of reticulocytes to erythrocytes, recent studies indicate that they are produced by different types of cells and are involved in promoting inter-cellular communication and antigen presentation. Here, we describe the isolation and characterization of exosomes from peripheral blood of BALB/c mice infected with the reticulocyte-prone non-lethal *Plasmodium yoelii* 17X strain. Importantly, proteomic analysis revealed the presence of parasite proteins in these vesicles. Moreover, immunization of mice with purified exosomes elicited IgG antibodies capable of recognizing *P. yoelii*-infected red blood cells. Furthermore, lethal challenge of immunized mice with the normocyte-prone lethal *P. yoelii* 17XL strain caused a significant attenuation in the course of parasitaemia, increased survival time, and altered the cell tropism to reticulocytes. These results were obtained also when the exosomes were isolated from a *P. yoelii*-infected reticulocyte culture indicating that reticulocyte-derived exosomes carry antigens and are involved in immune modulation. Moreover, inclusion of CpG ODN 1826 in exosome immunizations elicited IgG2a and IgG2b antibodies and promoted survival, clearance of parasites and subsequent sterile protection of 83% of the animals challenged with *P. yoelli* 17XL. To our knowledge, this is the first report of immune responses elicited by exosomes derived from reticulocytes opening new avenues for the modulation of anti-malaria responses.

## Introduction

Exosomes are 30–100-nm membrane vesicles formed by endocytosis of segments of the plasma membrane. The internalized segment generates multivesicular bodies (MVBs) containing small vesicles that are released as exosomes following fusion of the MVBs with the plasma membrane [1]. Initially described as a reticulocyte cargo-disposal mechanism for maturation to erythrocytes [2], exosomes are now known to be also secreted by many different types of cells, including dendritic cells, macrophages, B cells, and tumor cells [1], [3], [4]. Pioneering studies with exosomes secreted by Epstein-Barr virus (EBV)-transformed B-cells demonstrated stimulation of T-cell in an antigen-specific manner [5]. Since, the role of exosomes in antigen presentation and immune modulation has been amply demonstrated in different infections including *Salmonella*, *Mycobacterium*, and *Toxoplasma* where exosomes released by infected cells contain microbial proteins [1], [6]. The role of exosomes in antigen presentation in malaria, however, has not been described.

In addition to exosomes, other vesicles termed microparticles (MPs) circulate in blood [7]. MPs should not be confounded with exosomes as MPs originate by budding or shedding from the plasma membrane as opposed to fusion of the MVBs with the plasma membrane. Moreover, MPs are heterogeneous in shape and bigger in size (100–1000 nm) and present different protein composition [8], [9]. Of note, MPs have been described associated with malaria pathology both in human and rodent models [10]. Indeed, production of MPs by parasitized red blood cells with implications in malaria immune responses and inflammation has been recently described [11], [12].

Exosomes were originally described in reticulocytes where they allow remodeling of the plasma membrane in the maturation to erythrocytes by eliminating specific proteins [2]. Remarkably, reticulocytes are the cells preferentially, if not exclusively, invaded by different malaria parasites such as *Plasmodium vivax* [13] and the reticulocyte-prone non-lethal *Plasmodium yoelii* 17X strain [14]. We thus hypothesized that reticulocyte-derived exosomes (*rex*) in such infections, in addition to their role as cargo-disposable machinery, should contain parasite proteins involved in immune modulation. Here, we describe the presence of parasite proteins in exosomes obtained from experimental infections of BALB/c mice infected with *P. yoelii* 17X (*ex*Py). Moreover, we show that immunization of mice with *ex*Py results in modulation of the immune response to *P. yoelii* infection. In addition, when combined with cytosine-phosphate-guanosine oligodeoxynucleotides (CpG-ODN), immunization of mice with *rex*Py elicited IgG2a and IgG2b antibodies and protected mice from a lethal challenge. This is the first report of a role in immune modulation of reticulocyte-derived exosomes opening new avenues in the modulation of anti-malarial responses.

## Results

### Peripheral blood exosomes from *P. yoelii* 17X infections contain parasite proteins

**Figure 1:**
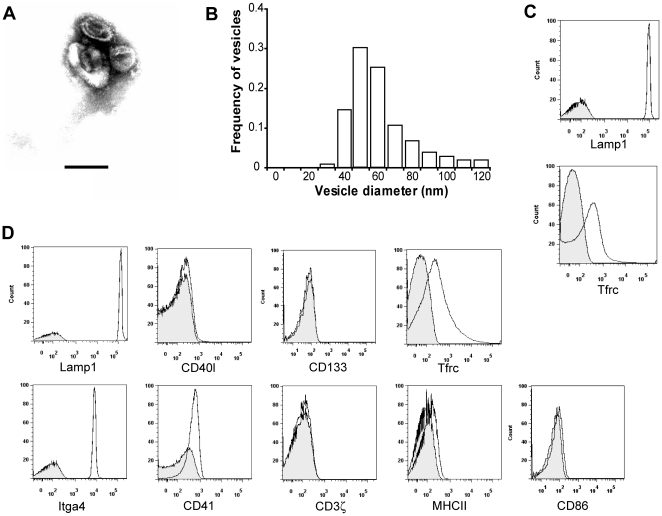
Characterization of exosomes from the peripheral blood of BALB/c mice infected with *P. yoelii* 17X. (**A**) Electron microscope image of negatively stained exosomes from plasma. Scale bar represents 100 nm. (**B**) Histogram of exosome size distribution was measured in ultramicrographs. X values represent the centre of an interval. (**C, D**) Characterization of surface markers present on blood-derived exosomes. Exosomes isolated from blood of infected mice were coated on latex beads, labeled with antibodies and detected by FACS analysis. (**C**) Histograms of representative marker of exosomes (Lamp1) and marker enriched in reticulocyte-derived exosomes (Tfrc) isolated by ultracentrifugation and sucrose-gradient purification. (**D**) Representative staining markers of exosomes (Lamp1), reticulocyte-derived exosomes (Tfrc, Itga4) and exosomes derived from T-cells (CD3ζ), APCs (MHC II, CD86), and platelets (CD41) performed on exosomes purified by ultracentrifugation and filtration. Histogram for the CD40l (microparticles) and CD133 (membrane particle) are also shown. Controls (tinted histograms) were performed with isotype controls or directly coupled secondary Abs.

To determine whether exosomes derived from malaria-infected mice contain parasite proteins, we purified exosomes from peripheral blood of BALB/c mice infected with *P. yoelii* 17X at approximately two weeks post-infection (p.i.), when reticulocytosis reached 60–90%. Electron microscopy (EM) analysis revealed a homogeneous population of vesicles whose cup-shape (Figure 1A) and size (median of 56.8 nm diameter) (Figure 1B) were consistent with previous descriptions of exosomes [15]. To better characterize these vesicles, they were coated on latex beads, stained with a panel of FITC-labeled or PE-labeled antibodies and analyzed by flow cytometry. Of note, vesicles isolated both by sucrose cushion (Figure 1C) as well as by ultracentrifugation/filtration (Figure 1D) display similar staining profiles for the proteins analyzed. CD107a (Lamp1) was found in vesicles isolated from blood of infected mice indicating that they were originated from an internal compartment and were not plasma membrane fragments. The presence of Lamp1 together with no presence of the microvesicle marker CD40l or membrane particle marker CD133 [8] indicates that our preparation of vesicles are mainly exosomes (Figure 1D). To determine whether the exosomes can come from different origins, we analyzed the presence of cell-specific molecules. Exosomes from infected blood showed the presence of CD71 (Tfrc) and Itga4 (integrin α4), molecules that were previously described in reticulocyte-derived exosomes [16]. Moreover, low staining for Antigen presenting cell (APC) markers class II MHC and CD86 and the platelet-specific marker CD41 (GpIIb) [17], [18] were also found on exosomes obtained from plasma. In addition, we could not find staining for the T-cell marker CD3ζ [17], [19], further indicating that vesicles obtained from these BALB/c–*P. yoelii* 17X infections are exosomes mostly originating from reticulocytes.

Next, we determined the proteome of exosomes by liquid chromatography-tandem mass spectrometry (LC-MS/MS). Results confirmed that exosomes purified from plasma of BALB/c mice infected with *P. yoelii* 17X contain many of the proteins most frequently found in exosomes such as Hsp8/Hsp90, Actb, Eno1, Anxa2, Ywhaz, Msn, Cfl1 and Actg1 [18] (Tables S1, S2). Moreover, a large number of proteins previously described in *rex* (Hsc70, CD47, aquoporin1 and Tfrc) [20]–[22] were also identified in our proteomic analysis. In contrast, no markers of MPs (CD40l) or apoptotic vesicles (Histones) [23] were found in the 100,000×g pellet. Remarkably, proteomic analysis revealed the presence of several *P. yoelii* antigens in exosomes (Table 1, S2). These parasite antigens included serine-repeat antigens, merozoite surface proteins 1 and 9, enzymes, proteases, heat-shock proteins, and hypothetical proteins. Altogether, these results showed that peripheral blood exosomes obtained from infections of BALB/c mice with *P. yoelii* 17X contain parasite proteins likely derived from infected reticulocytes.

**Table 1:** *P. yoelii* 17X proteins identified in peripheral blood exosomes.

Accession number	Description	Num. unique peptides	Num. spectra	Xcorr sum*	Min. peptide probability
PY00291	SERA 3	22	47	87,127	8,75E-11
PY00292	Papain family cysteine protease putative	18	36	70,545	3,75E-09
PY02883	merozoite surface protein 9 precursor putative	10	20	37,951	9,73E-05
PY05999	octapeptide repeat antigen	9	15	31,953	5,02E-05
PY03885	L lactate dehydrogenase	9	25	38,922	2,21E-10
PY05748	merozoite surface protein 1 precursor	9	14	33,698	7,54E-07
PY00427	3 nucleotidase/nuclease	7	14	27,502	8,52E-05
PY00293	Papain family cysteine protease putative	7	10	25,306	0,000569
PY04614	heat shock protein 60	6	8	20,313	4,49E-06
PY02351	Y13180 multicatalytic endopeptidase	5	10	19,382	3,76E-07
PY01759	hypothetical protein	3	4	8,462	1,93E-05
PY06307	hypothetical protein	3	6	11,238	1,98E-11
PY03639	cell division cycle protein 48 homolog	3	4	11,588	0,000967
PY04190	proteasome subunit alpha Type 6 B	3	5	8,93	4,95E-05
PY03212	proteasome beta subunit putative	3	3	11,273	1,32E-05
PY00275	hypothetical protein	2	3	7,002	0,000364
PY06203	blood stage membrane protein Ag 1	2	3	6,878	0,000256
PY03709	Fructose bisphosphate aldolase class I	2	3	5,899	0,000107
PY03625	secreted blood stage antigen pAg 3	2	5	7,153	0,000172
PY06158	heat shock protein 70	2	5	7,282	3,55E-06
PY01014	retinitis pigmentosa GTPase regulator like	2	4	9,099	2,83E-06
PY06644	enolase	2	2	7,82	0,000551
PY00267	proteasome subunit alpha type 1	2	4	6,468	2,06E-05
PY03280	glyceraldehyde 3 phosphate dehydrogenase	2	2	6,064	1,14E-06
PY00622	rhoptry associated protein 1	1	2	3,592	3,04E-05
PY06837	putative T complex protein beta subunit	1	1	3,733	9,08E-05
PY06834	yir4 protein	1	1	3,588	0,00064
PY06423	hypothetical protein	1	1	4,281	0,000742
PY06767	proteosome PSMB5/8 protein	1	2	4,167	1,31E-07
PY04294	hypothetical protein	1	2	3,763	0,000269
PY05295	hypothetical protein	1	2	4,502	5,97E-05
PY03834	AhpC/TSA family putative	1	2	5,318	1,52E-12

*Xcorr sum – Sum of the cross-correlation scores of individual peptides from each identified protein.

### Immunization with peripheral blood exosomes from *P. yoelii 17X* infections elicits parasite-specific immune humoral responses and modulates the course of infection upon lethal challenge

**Figure 2:**
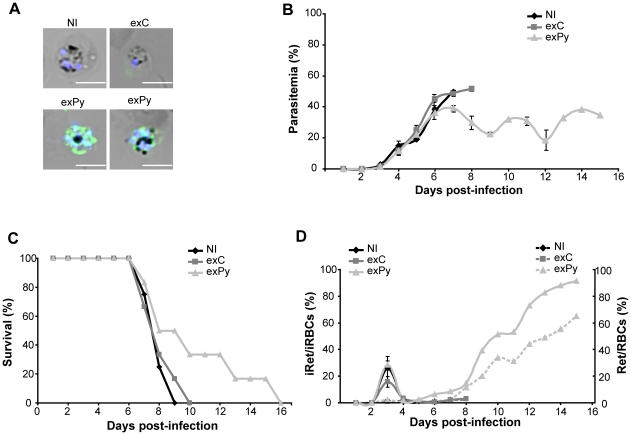
Exosomes from blood of *P. yoelii* 17X-infected mice modulate the course of *P. yoelii* 17XL infection. (**A**) Humoral IgG response after exosomal immunization of BALB/c mice. Bright-field and fluorescence image projections of immune sera recognizing 17XL-pRBCs. The images correspond to sera of representative mice. One non-immunized (NI) mouse, one mouse immunized with exosomes from control animals (exC) and two mice immunized with exosomes from mice infected with the *P. yoelii*17X non-lethal strain (exPy). Staining for mouse IgG (green) and DNA (blue) is shown. Scale bar represents 5 µm. (**B**) Time-course parasitaemia (mean±SD), (**C**) survival curve, (**D**) reticulocytosis (right axis, dotted line) and percentages of infected reticulocytes (left axis, continuous line) after 5×10^5^–10^6^ *P. yoelii* 17XL infections of groups of BALB/c mice previously immunized with exosomes from control animals (exC) (n = 6) and exosomes from mice infected with the *P. yoelii* 17X non-lethal strain (exPy) (n = 6). Non-immunized (NI) mice (n = 4) were untreated. Data correspond to 3 independent experiments. (**B**) *P. yoelii* 17X mean peak level of parasitemia is 50.3%±1.6% for NI, 51.77%±1.02% for exC and 39.14%±2.17% for exPy. The reduction was evaluated by analysis of variance and was statistically significant (P<0.001). (**C**) Differences in the survival curves between NI and exPy (P<0.05) are statistically significant (Log-rank (Mantel-Cox Test)).

To investigate whether peripheral blood exosomes derived from *Plasmodium*-infected mice could induce parasite-specific immune responses, we immunized mice with exosomes obtained from mice infected with the non-lethal *P. yoelii* 17X strain (exPy) and non-infected animals (exC) at days 0 and 20. Twenty days after the second immunization, sera were tested by immunofluorescence (IFA) for the presence of anti-*P. yoelii* antibodies. As shown in Figure 2A, immunizations with exPy elicited IgG antibodies capable of specifically recognizing infected RBCs. To further evaluate these immune responses, mice immunized with exC and exPy were challenged twenty days later with 5×10^5^–10^6^ *P. yoelii* 17XL lethal parasites. Daily examination of the animals and blood smears by Giemsa staining revealed an attenuation in the course of parasitaemia (Figure 2B) and higher survival times (Figure 2C) only in mice immunized with exPy. In addition, these mice had a significant increase of reticulocytosis (from 1–2% up to 65%) as well as a change in the parasite's cell tropism to reticulocytes (Figure 2D). This change in cell tropism does not appear to be simply the result of an increase in reticulocytosis as the percentage of circulating normocytes did not fall below 45%; yet, close to 90% of infected cells were reticulocytes. More importantly, these results demonstrated that peripheral blood exosomes in this rodent malaria model induced immune responses which modulated the course of infection.

### Reticulocyte-derived exosomes from infections are responsible for immune modulation, reticulocytosis and change of cell-tropism

**Figure 3:**
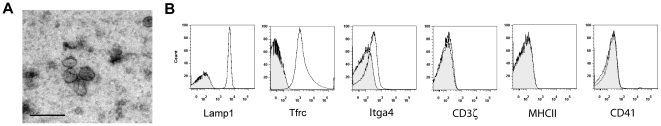
Characterization of exosomes isolated from supernatants of *in vitro* cultured *P. yoelii* 17X-infected reticulocytes. (**A**) EM image of negatively stained reticulocyte-derived exosomes. Scale bar represents 200 nm. (**B**) Characterization of surface markers present on reticulocyte-derived exosomes by FACS analysis. A total of 5 µg exosomes prepared from reticulocytes infected with *P. yoelii* 17X were coated on latex beads and labeled with Abs detectable by FACS analysis. Histograms of representative stainings for different vesicle markers are shown. Controls (tinted histograms) were performed with isotype controls or directly coupled secondary Abs.

Exosomes obtained from the blood of BALB/c mice infected with *P. yoelii* 17X are likely to be originated from reticulocytes; yet, exosomes from other cells capable of antigen presentation, such as dendritic cells could contribute to the final pool of exosomes. To exclude this possibility, isolated reticulocytes from blood of *P. yoelii*-infected mice were cultured for 24 hours and exosomes purified from supernatants by ultracentrifugation and filtration. Analysis of vesicles released by these cells by EM confirmed their purity and revealed a size and morphology compatible with exosomes (Figure 3A). Moreover, flow cytometry analysis of *rex* bound to aldehyde/sulfate beads revealed the expression in the surface of Lamp1, Tfrc, and Itga4 markers but not of MHC II, CD3ζ or CD41, confirming their origin from reticulocytes (Figure 3B). *In vitro*-purified *rex* from infections (rexPy) or from anemic mice (rexC) [21] were then used to immunize new groups of mice at 20-day intervals with two i.v. doses of 5 µg of *rex*Py or *rex*C. Nonimmunized mice (NI) were untreated. Twenty days after the second immunization, all mice were infected with 5×10^5^ *P. yoelii* 17XL lethal parasites. Noticeably, we observed the same results as those observed in immunizations with exosomes from peripheral blood, namely higher survival times, increase in reticulocytosis (from 1–2% up to 60%) and infection preference for reticulocytes in mice immunized with exPy (Figure S1). Thus, *rex* from infections contain parasite proteins and are involved in modulation of immune responses upon infection.

### Immunization with *rex* combined with CpG-ODN elicits protective immune responses

**Figure 4:**
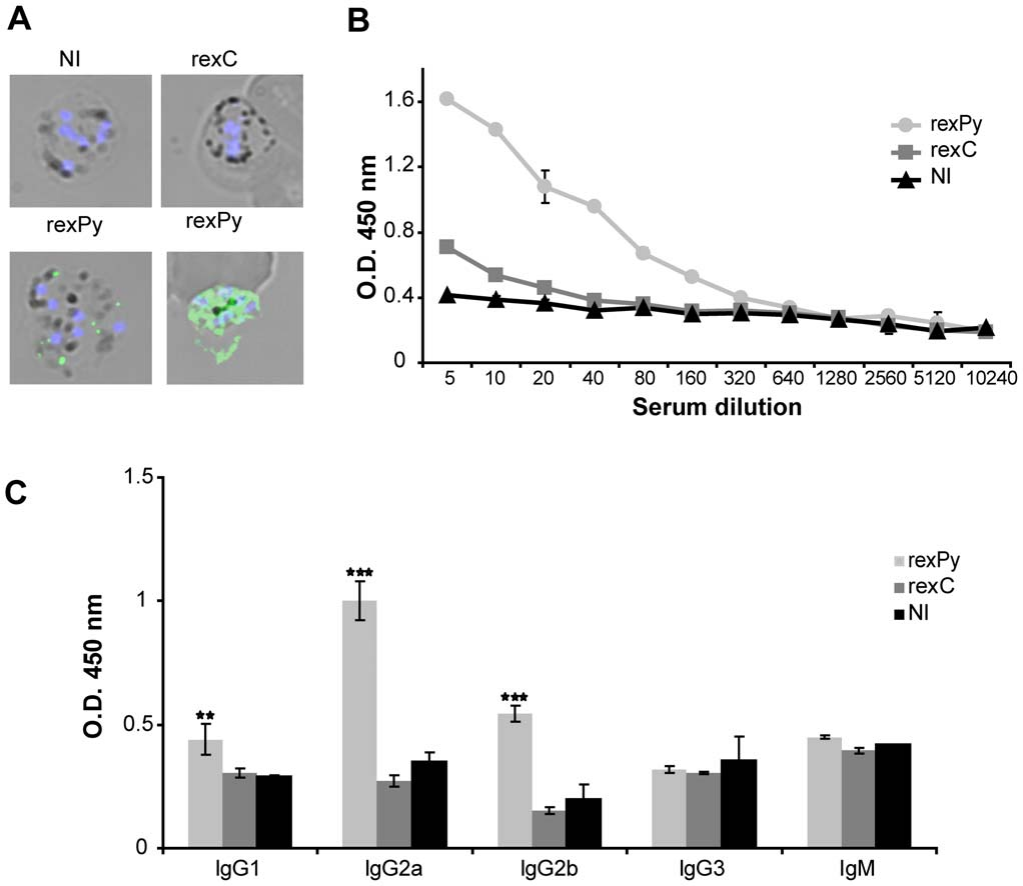
Humoral response induced by immunization of BALB/c mice with reticulocyte-derived exosomes. Humoral response after immunization of BALB/c mice with exosomes plus CpGODN-1826. (**A**) IgG responses against *P. yoelii*-infected RBCs. Bright-field and fluorescence image projection of immune sera recognizing 17XL-pRBCs. Staining for mouse IgG (green) and DNA (blue) is shown. NI, nonimmunized mice sera; *rex*C, sera from mice immunized with reticulocyte-derived exosomes; *rex*Py, sera from two different mice immunized with *P. yoelii*-infected reticulocyte-derived exosomes (**B,C**) Antibody responses induced by immunization detected by ELISA against *P. yoelii* antigen. (**B**) IgG response in immunized mice. Data show results with serial dilution of pooled sera (*n* = 6/group), corresponding to 3 independent experiments collected 20 days after the second immunization. (**C**) Antibody isotype response in pooled sera (n = 6) of mice immunized with exosomes. Sera were diluted 1∶50 before use. Data were evaluated by analysis of variance versus NI (**P<0.01 and ***P<0.001) (Tukey *post hoc* test).

CpG oligodeoxynucleotide (CPG-ODN) is a potent immunostimulator proving beneficial in protection against rodent malaria [24]. We thus tested the immune enhancement effect of CpG-ODN on immunization with *rex*. To do so, BALB/c mice were immunized subcutaneously (s.c.) with 10 µg of *rex*C or *rex*Py combined with 10 µg CpG-ODN. Twenty days after the first immunization, mice were boosted s.c. with 5 µg of exosomes and forty days later sera from immunized mice were tested by IFA and ELISA for the presence of antibodies against *P. yoelii*. As shown in Figure 4, immunizations with *rex*Py, but not with *rex*C or nonimmunized mice, elicited IgG antibodies recognizing infected RBCs (Figure 4A). In addition, ELISA analysis demonstrated that elicited antibodies were predominantly of the IgG2a and IgG2b isotypes with no detected differences in the levels of IgG3 and IgM (Figure 4C).

**Figure 5:**
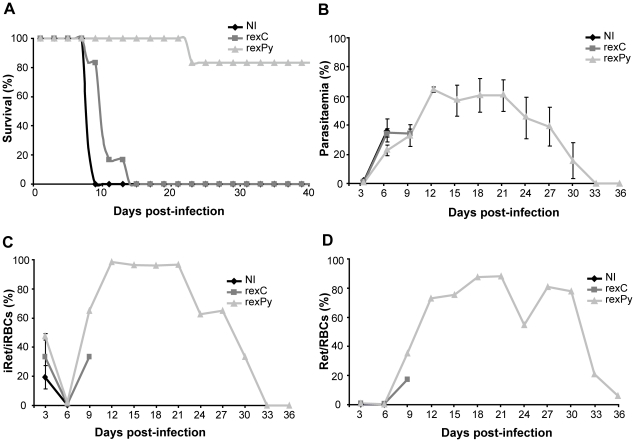
Immunization with exosomes from 17X-infected reticulocytes plus CpGODN-1826 protect mice from *P. yoelii* 17XL infection. (**A**) Survival curve, (**B**) time-course parasitaemia (mean±SD), (**C**) percentages of infected reticulocytes and (**D**) reticulocytosis after 5×10^5^ *P. yoelii* 17XL infections of groups of BALB/c mice previously immunized subcutaneously (s.c.) with CpG-ODN plus exosomes from control reticulocytes (*rex*C) (n = 6) or exosomes from reticulocytes infected with the *P. yoelii* 17X non-lethal strain (*rex*Py) (n = 6). Non-immunized (NI) mice (n = 6) were untreated. Data correspond to 3 independent experiments. (**A**) Differences in the survival curves between NI, rexC and rexPy (P<0.01) are statistically significant (Log-rank (Mantel-Cox Test).

Next, immunization with *rex*Py in combination with CpG-ODN was tested for the ability to protect mice against a lethal challenge with *P. yoelii* 17XL. Significantly, 83% of the mice immunized with *rex*Py survived to a lethal infection (Figure 5A) showing concomitantly reticulocytosis (Figure 5D) and a change of cell-tropism to reticulocytes (Figure 5C). Of importance, mice which survived the primary infection showed a complete clearance of infection (Figure 5B) and remained immunoprotected, showing no detectable parasitaemia by Giemsa smears, after re-challenges with *P. yoelii* 17XL at days 74, 116, and 156 post first infection.

## Discussion

Reticulocyte-derived exosomes function as a cargo-disposal machinery of unwanted proteins and molecules in the maturation of reticulocytes to erythrocytes [2]. Noticeably, malaria parasites such as *P. vivax* and the *P. yoelii* 17X strain predominantly, if not exclusively, infect reticulocytes. Here, we describe for what we believe is the first time, that *rex* from experimental infection of BALB/c mice with the *P. yoelii* 17X strain contain parasite proteins and that in addition to their cargo-disposable role in differentiation to erythrocytes, *rex* have a role in modulating immune responses.

The function of *rex* has been less explored than the one of exosomes derived from antigen-presenting cells, B cells, or tumor cells. In fact, different studies have identified proteins associated with these vesicles [25] and this has been recently confirmed and expanded in a proteomic analysis of *rex* from rats [26]. Our proteomic data from exosomes isolated from blood of *P. yoelii* 17X-infected mice revealed the presence of proteins previously described in *rex* such as Hsc70, CD47, aquoporin1 and Tfrc [20], [22]. Moreover, we identified the Na^+^-independent neutral aminoacid transporter LAT-1 [27], a function that was originally described in *rex* [28]. Remarkably, our proteomic data also revealed the presence of 31 parasite proteins within or associated with exosomes obtained from reticulocyte-enriched blood of infected animals. These proteins include several antigens such as merozoite surface proteins 1 and 9, as well as blood stage surface antigens, a rhoptry-associated protein and a variant Yir protein. In addition, we identified 6 hypothetical proteins, 3 of them having GO annotations related to binding (PY06307), pathogenesis (PY06423), and immune response/antigen processing presentation (PY05295). Moreover, we found 9 enzymes related to proteolysis and metabolic processes. The function of these enzymes is yet to be determined but it is worth recalling that some of them seem to have moonlight functions in malaria [29]–[32]. In fact, we identified enolase (PY06644) which has been found in different locations suggesting non-glycolytic functions of this enzyme [33] and which upon immunization as a recombinant protein partially protects mice against *P. yoelii* [33]. While proteomic analysis of *rex*Py, *per se*, remains to be determined, this data unequivocally demonstrates the presence of malaria proteins in exosomes derived from peripheral blood in this reticulocyte-prone rodent malaria model.

Peripheral blood contains other type of vesicles termed microparticles (MPs) which have been described in malaria infections both in humans and animal models. Thus, MPs from platelets bind to erythrocytes transferring platelet antigens which are postulated to facilitate cytoadherence to brain tissues leading to cerebral malaria [34]. In addition, MPs have also been recently described in human falciparum and vivax infections [12], [35] and have been associated with acute vivax malaria symptoms [35]. Moreover, in rodent models, MPs derived from malaria infected RBCs have been recently shown to lead to inflammation through macrophage activation [11]. Noticeably, if shedding of these vesicles occurs during *P. yoelii* 17X infections, they did not contribute to our results since MPs were discarded during our exosome preparation (pellet after 15,000×g centrifugation, see methods). Moreover, flow cytometry revealed no significant amounts of CD40l, a MP marker, in our vesicle preparations. In addition, EM analysis allowed us to check for purity of our isolated exosomes thus discarding any preparation containing contamination with particles that differ from the cup-shape and size reported for exosomes.

Immunizations of BALB/c mice with exosomes derived from peripheral blood or from *rex* purified from in vitro cultures from infections, elicited IgG antibodies capable of recognizing *P. yoelii*-infected RBCs, induced reticulocytosis and changed the cell-tropism to reticulocytes of the normocyte-prone lethal *P. yoelii* 17XL strain upon infection. Moreover, when combined with CpG-ODN, immunizations with *rex*Py obtained from reticulocyte *in vitro* culture elicited the same responses as above in addition to conferring complete and long lasting protection in close to 85% of the mice tested. Of note, antibody responses in this lethal strain of *P. yoelii* are mainly suppressed during infections [36]; yet, these immunizations elicited IgG2a antibodies capable of recognizing *P. yoelii*-infected RBCs. This humoral response is likely associated with protection since it has been described that passive transfer of the IgG2a fraction of hyperimmune plasma can modulate parasitaemias of BALB/c mice infected with *P. yoelii* 17XL [37]. Remarkably, reticulocytosis, change of cell-tropism and protection mediated by IgG2a antibodies were reported upon immunizations of BALB/c mice with the *P. yoelii* 235-kDA antigen [38] and with the *P. yoelii* MSP8 protein [39]. Moreover, in this latter study, genome-wide global transcriptional analysis revealed the presence of up-regulated genes coding for parasite antigens also identified in our proteomic analysis such as MSP1, MSP9, rhopthry-associated proteins and blood stage surface antigens (pAgI and pAg3) [39]. Protection in all these reports seems depending on elicitation of IgG2a antibodies that putatively block entrance into mature red blood cells and which, upon infections with the lethal strain, would favor the survival of parasites entering reticulocytes. In turn, it is tempting to speculate that infected reticulocytes should release *rex* containing parasite proteins which act in inter-cellular communication and antigen presentation, thus facilitating reticulocytosis and the mounting of a protective and long-lasting immune response. In the absence of supporting experimental evidence, this remains to be demonstrated.

To our knowledge, the results presented here provide the first description of exosomes, not MPs, containing malaria proteins. Moreover, our study also shows that, when combined with CpG-ODN, *rex* from infections have the capacity of elicitation of protective and long-lasting immune responses against lethal infections in this rodent malaria model. While further experimentation is required to determine the exact role of *rex* during infections, *rex* represent a new player in immune response to malaria that could also be used as a vaccine tool against infections.

## Materials and Methods

### Ethics statement

All the animal studies were performed at the animal facilities of Hospital Clinic in Barcelona in accordance with guidelines and protocols approved by the Ethics Committee for Animal Experimentation of the University of Barcelona CEEA-UB.

### Mice and parasites

Female BALB/c mice, 6 to 8 weeks of age, were used throughout the study. The *P. yoelii* 17XL (lethal) and 17X (non-lethal) cloned lines were originally obtained from MR4 (http://www.mr4.org/). Infections were initiated by intraperitoneal (i.p.) injection of 10^5^–10^6^ pRBCs from the tail blood of donor mice at 5–10% parasitaemia. Parasitaemia was monitored by Giemsa staining of blood smears calculating the percentage of pRBCs over total RBCs in three optical fields of approximately 300 RBCs.

Reticulocytosis was induced by phlebotomy as previously described [21]. Blood was collected by cardiac puncture in anesthetized mice. Brilliant Cresyl Blue (BCB)-Giemsa staining was used to detect reticulocytes [40]. Briefly, five µl of mouse blood was mixed with an equal volume of 1% BCB in 0.65% sodium chloride and incubated at room temperature (RT) for 20 min. After incubation, smears were prepared and left to air dry. After fixation with methanol, the smears were counterstained with Giemsa. Percentage of reticulocytes over total RBCs was counted in different optical fields with, at least, 1000 RBCs.

### Purification of reticulocytes

Reticulocytes were obtained from mice blood collected in EDTA. We have used blood of BALB/c anemic mice and of mice infected with *P. yoelii* 17X strain at 20–30% parasitaemia. To isolate the reticulocytes, blood was centrifuged at 1000×*g* for 20 min. After two washes in phosphate-buffered saline (PBS), reticulocytes were purified by layering them on top of a Percoll/NaCl density gradient as previously described [21], [41]. Briefly, 5 ml of 1.096 g/mL of density Percoll solution was placed in 15 mL tubes and 2 mL of 1.058 g/mL Percoll were layered over. Thereafter, 2 mL of blood diluted in PBS to a final hematocrit of 50% were layered on top of each tube. Tubes were centrifuged at 250×g for 30 minutes at 4°C. Reticulocytes were collected from the interface of the two Percoll layers and washed twice with PBS. We routinely obtained 7±3×10^7^ reticulocytes per uninfected mice and 3.6±0.6×10^8^ reticulocytes per *P. yoelii* 17X-infected mice. Purified reticulocytes were cultured for 24 h at 37°C in DMEM, supplemented with 5 mM glutamine, 5 mM adenosine, 5 mM inosine, 5% fetal calf serum, 50 U/mL penicillin, and 50 µg/mL streptomycin at 1–3% hematocrit. To remove exogenous exosomes in the culture medium, the fetal calf serum was precentrifuged (100,000×g overnight) (o.n.).

### Exosomes

Exosomes were purified from the plasma of uninfected mice or mice infected with *P. yoelii* 17X by sequential centrifugations at 500×*g* for 30 min, 15,000×*g* for 45 min and 100,000×*g* for 2 h at 4°C (adapted from [17]). The final pellet was resuspended in PBS, filtered through a 0.22-µm membrane, and centrifuged at 100,000×*g* for 2 h at 4°C. The pellets were resuspended in PBS, and the protein content was determined by Bradford assay. Usually, we are able to obtain the equivalent of 5–15 µg of proteins in exosomes per ml of blood (7.8±2.4/uninfected mice, 12.7±1.9/*P. yoelii* 17X mice). Routinely, exosomes purity was assessed by ME analysis.

Exosomes from supernatant of culture were obtained with the same differential centrifugation method.

For some applications, exosomes were loaded in a 30% sucrose cushion (adapted from [15]). Briefly, exosome pellet from first centrifugation at 100,000×g was resuspended in 6 ml PBS and loaded on top of 1 ml sucrose/D_2_O solution (20 mM Tris, 30% sucrose, pH 7.4). Samples were centrifuged in a swinging bucket rotor at 100,000×g for 75 min. Approximately 0.75 ml of the cushion containing exosomes was collected, diluted with PBS and centrifuged at 100,000×g for 90 min. Washed exosomes were then resuspended in PBS and protein content was measured using Bradford assay.

### Electron microscopy

Electron microscope analysis was performed with exosomes fixed in 2% paraformaldehyde (PFA) o.n. at 4°C. Fixed exosomes were deposited onto formvar grids for 20 min. Grids were then fixed in glutaraldehyde 1% for 5 min, washed in distilled water and negatively stained for 5 min with a solution of uranyl-oxalate (pH = 7) and for 10 min at 4°C with uranyl acetate(4%)-methyl cellulose (2%). After thoroughly drying, grids were observed with a JEOL 1010 transmission electron microscope. Micrographs were used to quantify the diameter of exosomes.

### Flow cytometry

Five micrograms of purified exosomes were incubated with 10 µl of 4 µm-diameter aldehyde/sulfate beads (4-µm beads, Invitrogen) for 15 min at room temperature (RT). PBS was added to a final volume of 1 ml and exosome-beads were incubated with rotation overnight at 4°C. After adsorbing exosomes, beads were subjected to glycine (100 mM final for 30 min at RT), followed by the addition of 0.5% BSA in PBS to saturate any remaining free binding sites on the beads. Coated beads were incubated with different antibodies (some directly conjugated to fluorescent dyes) diluted in PBS/0.5% BSA 30 min at 4°C. After washing, samples were incubated as needed with fluorescent-labeled secondary antibodies diluted in PBS/0.5% BSA for 30 min at 4°C. Samples were analyzed by flow cytometry using a FACSCanto instrument. A minimum of 5×10^5^ single beads per sample was examined.

### Proteomic analysis of exosomes

Exosomal preparations were dissolved in 8 M urea 0.4 M NH_4_HCO_3_, reduced with 5 mM dithiothreitol for 15 min at 50°C, alkylated with 10 mM iodoacetamide for 30 min, at room temperature, and the reaction was diluted to obtain a final concentration of 1 M urea. After digestion with trypsin for 16 h, samples were desalted using POROS R2 ziptips [42], redissolved in 0.1% formic acid (FA), loaded in a strong-cation exchange trap column (5 µL, Optimized Technologies) and eluted by injecting increasing concentrations of NaCl (0, 25, 50, 100, 200, and 500 mM NaCl in 0.5% FA/2% acetonitrile (ACN)) using the autosampler, which is part of the 1D Plus nanoHPLC system (Eksigent). Eluted peptides were trapped and washed (0.5% FA in 2% ACN) in a laboratory-made C18 column (1 cm, 75 µm, Phenomenex Luna C18, 5 µm); separation was achieved in a capillary C18 column (20 cm, 75 µm, Phenomenex Luna C18, 5 µm), using a linear acetonitrile (ACN) gradient of 5–40% B solvent over 100 min (solvent A: 5% ACN/0.1% FA, solvent B: 80% ACN/0.1% FA). Peptides were analyzed online on a LTQ XL/ETD (Thermo Fisher Scientific) mass spectrometer equipped with a Triversa Nanomate (Advion) nanospray source. Full scan MS spectra were collected from 400–1700 m/z range and the ten most intense ions were selected for fragmentation twice, before dynamically excluding for 1 min. MS/MS spectra were converted to DTA files and submitted to database search using Sequest. The search parameters were: (i) full tryptic digestion with 1 missed cleavage site allowed; (ii) 2 Da of peptide mass tolerance; and (iii) methionine oxidation and cysteine carbamidomethylation as variable and static modifications, respectively. The database (129,706 total sequences) was built with the forward and reverse sequences of *P. yoelii* v5.5 (http://plasmodb.org), in addition to possible murine (IPI mouse v3.58) and human (keratins derived from IPI human v3.58) contaminant sequences downloaded from http://www.ebi.ac.uk/IPI/IPIhelp.html. Data were filtered with DCn≥0.05, peptide probability ≤0.05 and Xcorr ≥1.5, 2.4 and 3.2 for 1+, 2+, and 3+ charged peptides, respectively. After assembling the peptides into proteins and redundant proteins into protein groups, the dataset was further filtered with Xcorr sum (sum of Xcorr of unique peptides for each protein) ≥3.5 to obtain a false-discovery rate of 0.8% at protein level.

### Immunizations and challenge

For immunizations, mice were injected intravenously with two doses of 5 µg of exosomes obtained by ultracentrifugation and filtration and resuspended in PBS at days 0 and 20. Non-immunized mice were untreated. Endotoxin level of exosomes was <0.005EU/µg (determined by kinetic chromogenic LAL assay, Genescript). Immunized animals were challenged twenty days after the second immunization with 5×10^5^–10^6^ *P. yoelii* 17XL parasites, and parasitaemia and reticulocytosis were followed daily using Giemsa-stained blood smears.

For CpG immunizations, mice were injected subcutaneouslly (s.c.) with 10 µg of exosomes and 10 µg CpG ODN-1826. Endotoxin level of exosomes was <0.005EU/µg (determined by kinetic chromogenic LAL assay, Genescript). Twenty days after, mice were immunized with 5 µg of exosomes. Twenty days after the second immunization, mice were infected with 5×10^5^ *P. yoelii* 17XL. Surviving animals were re-infected three times with *P. yoelii* 17XL at days 74, 116 and 156 after the first infection. Parasitaemia was followed using Giemsa-stained blood smears.

### Antibody responses

Humoral responses elicited by exosomes were studied by immunofluorescence and ELISA using mouse sera collected 20 days after immunizations and tested for parasite recognition. IFA assays were performed on *P. yoelii* 17XL-infected blood smears fixed with cold methanol for 2 min and air-dried before blocking with 5% BSA/PBS for 30 min at room temperature (RT). Slides were incubated with mouse sera diluted 1/10 in 0.5% BSA/PBS o.n. at 4°C followed by incubation for 1 h at RT. Reactive IgGs were detected using an anti-mouse IgG antibody conjugated to Alexa Fluor 488 (Invitrogen) at 1∶200 dilution for 1 h at RT. After washing, nuclei were stained with DAPI (Invitrogen, 5 mg/mL) for 7 min at RT. Sera recognizing pRBCs were imaged for bright field and fluorescence using a Leica SP5 microscope fitted with an inverted 63× oil objective. Sera from control non-immunized mice and mice immunized with exosomes from uninfected animals were used as negative controls.

Serum antibody levels were determined by an enzyme-linked immunosorbent assay (ELISA) against *P. yoelii* antigen, with pooled sera (n = 6 mice per group). MaxiSorb immunoplates (Nunc) were coated with 200 ng of parasite antigen, obtained as described [43], in carbonate-bicarbonate buffer pH 9.6, overnight at 4°C. Wells were blocked with 100 µl 5% BSA in coating buffer at 37°C for 3 h. After four washes with 0.05% Tween in PBS, two-fold serial dilutions of serum samples were added in 1% BSA in PBS. After incubation for 1 h at 37°C, wells were washed and incubated with peroxidase-conjugated anti-mouse IgG. For isotype analysis bound antibodies were detected with peroxidase conjugated anti-mouse (IgG1, IgG2a, IgG2b, IgG3, or IgM) antibodies (Abcam). After incubation for 1 h, wells were washed and incubated with 50 µl TMB substrate (Pierce) for 15 min. The reaction was stopped with 50 µl 1N sulfuric acid per plate well. Absorbance was measured at 450 nm.

## Supporting Information

Figure S1 **Immunization with exosomes from 17X-infected reticulocytes modulated the course of** ***P. yoelii*** **17XL infection.** (**A**) Survival curve, (**B**) time-course parasitaemia (mean±SD), (**C**) percentages of infected reticulocytes and (**D**) reticulocytosis after 5×10^5^ *P. yoelii* 17XL infections of groups of BALB/c mice previously immunized intravenously (i.v.) with 5 µg exosomes from control reticulocytes (*rex*C) (n = 5) or 5 µg of exosomes from reticulocytes infected with the *P. yoelii* 17X non-lethal strain (*rex*Py) (n = 5). Non-immunized (NI) mice (n = 5) were untreated. Data correspond to 2 independent experiments. (**A**) Differences in the survival curves between NI and rexPy (P<0.05) are statistically significant (Log-rank (Mantel-Cox Test).
(TIF)

Table S1 **Mouse proteins** identified in exosomes derived from mice infected with non-lethal strain of *Plasmodium yoelii* 17X.
(DOC)

Table S2 Detailed information about peptide Identification of proteins from exosomes derived from mice infected with non-lethal strain of *Plasmodium yoelii* 17X.
(XLS)
