# Supplementary figures and images for: Exosomes from Plasmodium yoelii-Infected Reticulocytes Protect Mice from Lethal Infections
Source: PLoS One. 2011 Oct 26;6(10):e26588. doi: 10.1371/journal.pone.0026588 (PMC3202549; doi:10.1371/journal.pone.0026588)

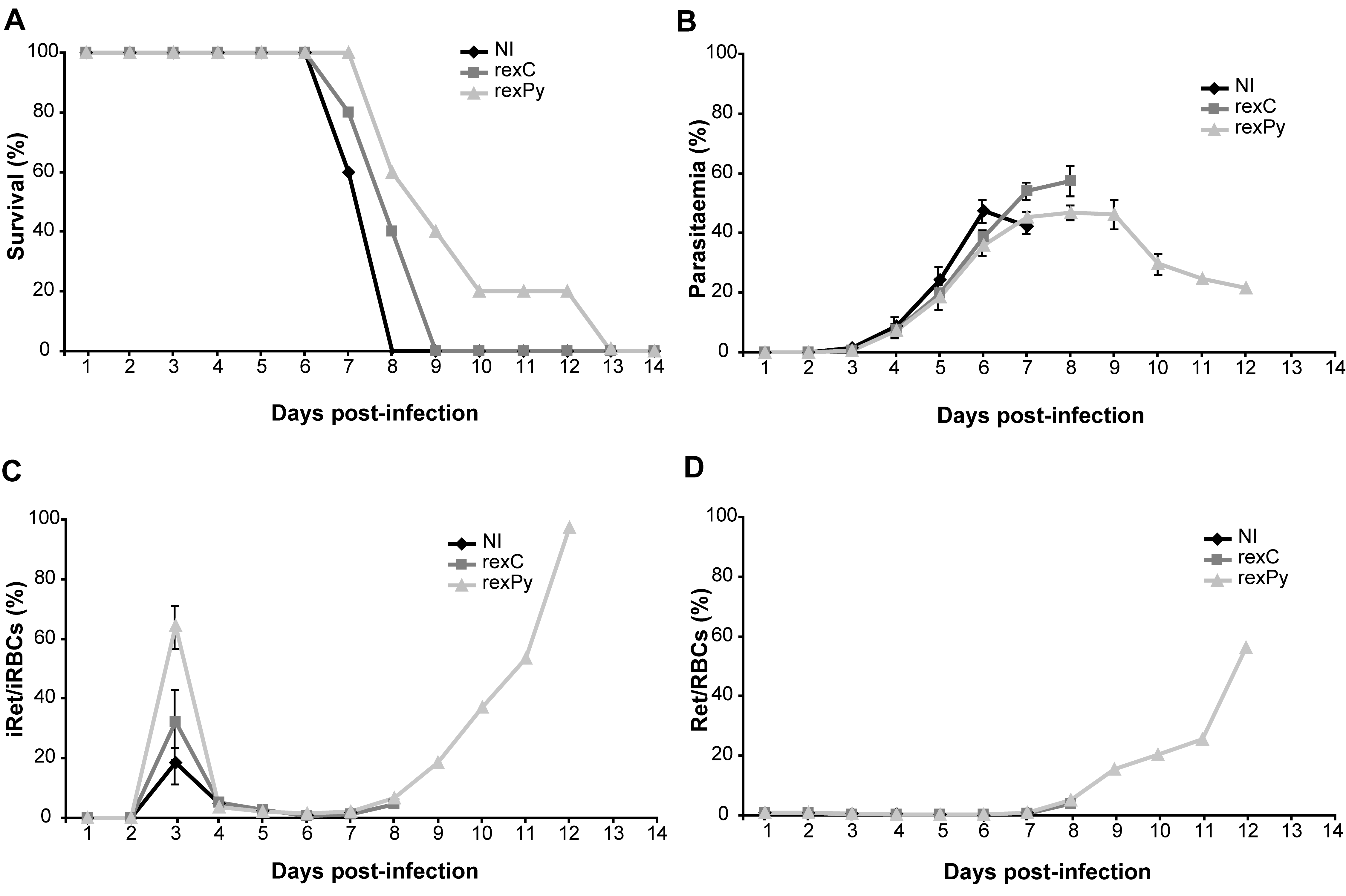

Supplement: Figure S1 — Immunization with exosomes from 17X-infected reticulocytes modulated the course of P. yoelii 17XL infection. (A) Survival curve, (B) time-course parasitaemia (mean±SD), (C) percentages of infected reticulocytes and (D) reticulocytosis after 5×10^5^ P. yoelii 17XL infections of groups of BALB/c mice previously immunized intravenously (i.v.) with 5 µg exosomes from control reticulocytes (rexC) (n = 5) or 5 µg of exosomes from reticulocytes infected with the P. yoelii 17X non-lethal strain (rexPy) (n = 5). Non-immunized (NI) mice (n = 5) were untreated. Data correspond to 2 independent experiments. (A) Differences in the survival curves between NI and rexPy (P<0.05) are statistically significant (Log-rank (Mantel-Cox Test). (TIF) [file pone.0026588.s001.tif]
