# Supplementary material for: Exosomes from Plasmodium yoelii-Infected Reticulocytes Protect Mice from Lethal Infections
Source: PLoS One. 2011 Oct 26;6(10):e26588. doi: 10.1371/journal.pone.0026588 (PMC3202549; doi:10.1371/journal.pone.0026588)
Supplement: Table S1 — Mouse proteins identified in exosomes derived from mice infected with non-lethal strain of Plasmodium yoelii 17X. (DOC) [file pone.0026588.s002.doc]

**Table S1** Mouse proteins identified in peripheral blood exosomes derived from mice infected with non-lethal strain of *Plasmodium yoelii* 17X.

| Identified protein | Gene | Num. peptides | Num.  spectra | Xcorr sum* | Peptide probability |
| --- | --- | --- | --- | --- | --- |
| Alpha 2 macroglobulin | Pzp | 72 | 973 | 305,92 | 1,11E-07 |
| Complement component 3 | C3 | 63 | 167 | 224,82 | 4,57E-13 |
| Albumin | Alb | 48 | 1056 | 204,51 | 6,00E-09 |
| Transferrin | Trf | 42 | 444 | 172,86 | 3,70E-10 |
| Murinoglobulin 1 | Mug1 | 29 | 91 | 113,73 | 2,16E-08 |
| Serine protease inhibitor A3K | Serpina3k | 26 | 177 | 109,08 | 1,58E-07 |
| Fibronectin | Fn1 | 21 | 59 | 73,21 | 1,19E-07 |
| Ig heavy chain | Igh | 24 | 492 | 102,53 | 8,77E-07 |
| Ceruloplasmin | Cp | 21 | 42 | 73,71 | 9,62E-06 |
| Vitamin D_binding protein | Gc | 20 | 52 | 89,39 | 1,49E-08 |
| Transitional endoplasmic reticulum ATPase | Vcp | 20 | 36 | 72,96 | 5,40E-06 |
| Complement component 1 s | C1s | 19 | 56 | 79,98 | 5,35E-07 |
| Hemopexin | Hpx | 17 | 72 | 61.91 | 2,38E-06 |
| Trasnferrin receptor protein 1 | Tfrc | 17 | 29 | 57,41 | 3,93E-05 |
| 1300017J02Rik | Mica | 15 | 31 | 55,04 | 3,86E-06 |
| Hemoglobin subunit beta 2 | Hbb b2 | 13 | 70 | 48.28 | 7,33E-12 |
| Heat shock cognate 71 kDa protein | Hspa8/Hsc70 | 13 | 22 | 44,38 | 3,55E-06 |
| Hemoglobin subunit beta 1 | Hbb b1 | 13 | 150 | 54,60 | 1,85E-10 |
| Plasminogen | Plg | 13 | 23 | 48,49 | 9,99E-15 |
| Alpha 1 antitrypsin 1 2 | Serpina1b | 12 | 43 | 53,03 | 2,53E-05 |
| Complement C5 | Hc | 12 | 20 | 40,43 | 0,00017 |
| Antithrombin III | Serpinc1 | 12 | 18 | 40,77 | 0,000805 |
| Histidina rich glycoprotein | Hrg | 12 | 23 | 40,31 | 1,85E-05 |
| Complement C4 B | C4b | 11 | 19 | 45,90 | 5,78E-10 |
| 10_formyltetrahydrofolate dehydrogenase | Aldh1l1 | 12 | 18 | 42,82 | 1,81E-06 |
| Serine protease inhibitor A3M | Serpina3m | 12 | 58 | 53,04 | 1,58E-07 |
| Mannan_binding lectin serine protease 1 | Masp1 | 12 | 18 | 43,48 | 2,60E-06 |
| Ig kappa chain | Igk | 4 | 25 | 17,48 | 1,08E-07 |
| Esterase 1 | Es1 | 11 | 48 | 43,16 | 4,92E-07 |
| Apolipoprotein A I | Apoa1 | 11 | 29 | 34,58 | 0,000725 |
| Complement C1r | Cr1 | 11 | 28 | 43,04 | 3,24E-07 |
| Delta_aminolevulinic acid dehydratase | Alad | 10 | 22 | 37,48 | 4,43E-06 |
| Integrin alphaV | Itgav | 10 | 49 | 38,68 | 2,00E-06 |
| Alpha 2 macroglobulin P | A2m | 10 | 39 | 31,21 | 2,71E-05 |
| Fibrinogen gamma polypeptide | Fgg | 9 | 41 | 39,60 | 3,18E-07 |
| Serpina1b protein | Serpina1b | 9 | 34 | 35,83 | 2,53E-05 |
| Kininogen 1 | Kng1 | 9 | 19 | 31,60 | 1,49E-05 |
| Actin cytoplasmic | Actg1 | 9 | 20 | 32,70 | 0,000403 |
| Serine _or cysteine_ proteinase inhibitor clade A member 1c | Serpina1c | 9 | 39 | 37,79 | 8,67E-09 |
| Serine protease inhibitor A3N | Serpina3n | 9 | 35 | 38,44 | 2,88E-06 |
| Keratin_ type II cytoskeletal 1 | Krt1 | 9 | 27 | 27,24 | 0,000458 |
| Gelsolin | Gsn | 9 | 21 | 35,08 | 1,37E-07 |
| Talin 1 | Tln1 | 9 | 11 | 28,16 | 0,000105 |
| Mannose_binding protein C | Mbl2 | 8 | 68 | 35,22 | 2,15E-10 |
| Ferritin light chain 1 | Ftl1 | 8 | 47 | 35,22 | 6,52E-08 |
| Serpina1d Alpha 1 antitrypsin 1 4 | Serpina1d | 8 | 36 | 34,16 | 2,64E-06 |
| Apolipoprotein E | Apoe | 8 | 19 | 31,89 | 0,000144 |
| C 1 tetrahydrofolate synthase cytoplasmic | Mthfd1 | 7 | 11 | 24,64 | 2,92E-05 |
| complement factor B isoform 2 | Cfb | 7 | 14 | 26,51 | 1,76E-07 |
| Integrin alpha_IIb | Itga2b | 7 | 12 | 27,08 | 1,50E-10 |
| Ig lambda chain | Igl | 7 | 69 | 29,52 | 9,16E-05 |
| Ig j chain | Igj | 7 | 39 | 23,13 | 1,43E-06 |
| Olfactory receptor 164 | Olfr164 | 7 | 36 | 33,64 | 2,14E-09 |
| Complement C1q subcomponent subunit B | C1qb | 7 | 54 | 26,62 | 2,60E-08 |
| Proteasome subunit alpha type 6 | Psma6 | 7 | 12 | 25,3 | 2,80E-07 |
| Vitronectin | Vtn | 7 | 15 | 25,83 | 1,21E-07 |
| Predicted gene_ 382044 | 382044 | 7 | 25 | 24,60 | 3,82E-05 |
| Actin, alpha, cardiac muscle 1 | Actc1 | 6 | 14 | 19,40 | 4,69E-06 |
| Hba a2 Hemoglobin subunit alpha | Hba a1 | 6 | 39 | 20,44 | 4,92E-08 |
| Itih1 protein | Itih1 | 6 | 10 | 26,67 | 1,13E-06 |
| Band 3 anion transport protein | Slc4a1 | 6 | 24 | 20,93 | 0,000385 |
| Keratin type I cytoskeletal 9 | Krt9 | 6 | 11 | 26,40 | 4,52E-08 |
| Afamin | Afm | 6 | 9 | 23,63 | 5,49E-09 |
| Proteasome subunit beta type1 | Psmb1 | 6 | 12 | 20,011 | 2,76E-08 |
| Sodium/potassium transporting ATPase subunit alpha 1 | Atp1a1 | 6 | 7 | 20,34 | 7,55E-06 |
| Proteasome subunit beta type 4 | Psmb4 | 6 | 9 | 21,14 | 5,77E-07 |
| Galectin 3 binding protein | Lgals3bp | 5 | 8 | 19,40 | 1,40E-09 |
| Complement component 4 binding protein | C4bp | 5 | 9 | 15,87 | 0,000125 |
| Filamin_ alpha | Flna | 5 | 7 | 19,66 | 9,70E-06 |
| Prothrombin Fragment | F2 | 5 | 9 | 16,54 | 0,000209 |
| Adenylyl cyclase associated protein 1 | Cap1 | 5 | 11 | 23,74 | 1,97E-08 |
| Complement factor I | Cfi | 5 | 15 | 17,04 | 5,08E-08 |
| Clusterin | Clu | 5 | 10 | 16,74 | 3,44E-08 |
| Alpha_2_antiplasmin | Serpinf2 | 5 | 8 | 14,66 | 1,74E-06 |
| Apolipoprotein A_IV | Apoa4 | 5 | 10 | 17,25 | 8,24E-08 |
| Complement component 1 q subcomponent A chain | C1qa | 5 | 20 | 17,65 | 4,55E-07 |
| Beta 2 glycoprotein 1 | Apoh | 5 | 7 | 15,25 | 1,61E-09 |
| Serum amyloid P component | Apcs | 5 | 19 | 20,46 | 1,00E-30 |
| Plasma protease C1 inhibitor | Serping1 | 5 | 9 | 18,30 | 1,50E-06 |
| Mannan_binding lectin serine protease 2 | Masp2 | 5 | 12 | 20,00 | 2,37E-06 |
| Integrin beta 3 precursor | Itgb3 | 5 | 8 | 16,92 | 1,10E-06 |
| Corticosteroid binding globulin | Serpina6 | 5 | 11 | 22,66 | 2,03E-07 |
| Leukocyte common antigen | Ptprc | 5 | 8 | 15,19 | 0,000886 |
| H_2 class I histocompatibility antigen_ K_D alpha chain | H2_K1 | 5 | 10 | 19,39 | 1,21E-07 |
| Major vault protein | Mvp | 5 | 6 | 14,92 | 6,07E-07 |
| Ferritin heavy chain | Fth1 | 5 | 8 | 17,01 | 2,68E-08 |
| Eukaryotic translation elongation factor 1 delta isoform b | Eef1 | 4 | 7 | 13,15 | 1,34E-06 |
| Erythrocyte band 7 integral membrane protein | Stom | 4 | 7 | 17,94 | 9,52E-08 |
| Ficolin 1 | Fcna | 4 | 6 | 13,66 | 2,36E-05 |
| Complement componet factor H | Cfh | 4 | 8 | 13,79 | 1,72E-07 |
| Carboxypeptidase N subunit 2 | Cpn2 | 4 | 8 | 14,10 | 0,000203 |
| Apolipoprotein B precursor | Apob | 4 | 6 | 12,95 | 0,000424 |
| Corticosteroid binding globulin Fragment |  | 4 | 8 | 18,55 | 2,37E-06 |
| Proteasome subunit alpha type 1 | Psma1 | 4 | 7 | 13,09 | 0,000381 |
| Glutathione peroxidase 3 | Gpx3 | 4 | 8 | 10,62 | 0,000114 |
| H2 class I histocompatibility antigen Q10 alpha chain | H2 Q10 | 4 | 6 | 14,59 | 2,04E-07 |
| Ras related protein Rap 1b | Rap1b | 4 | 6 | 13,47 | 0,000229 |
| Fga fibrinogen alpha polypeptide isoform 2 | Fga | 4 | 8 | 13,62 | 4,70E-05 |
| Fibrinogen beta chain | Fgb | 4 | 11 | 18,00 | 0,000957 |
| Integrin beta 2 | Itgb2 | 4 | 7 | 17,87 | 1,47E-06 |
| Proteasome subunit beta type 8 | Psmb8 | 4 | 5 | 14,75 | 0,000464 |
| Carboxypeptidase N catalytic chain | Cpn1 | 4 | 6 | 12,39 | 1,21E-05 |
| Apolipoprotein A II | Apoa2 | 4 | 7 | 17,39 | 0,000142 |
| Proteasome subunit alpha type 4 | Psma4 | 4 | 8 | 15,78 | 2,53E-12 |
| Proteasome subunit beta type 3 | Psmb3 | 4 | 6 | 16,40 | 1,33E-05 |
| 14_3_3 protein zeta/delta | Ywhaz | 4 | 8 | 20,08 | 3,14E-10 |
| H2 MHC class I H_2Dp _Fragment | H2 Q2 | 3 | 5 | 13,02 | 9,57E-05 |
| Isoform 1 of Basigin | Bsg | 3 | 5 | 8,53 | 2,08E-04 |
| H2 class I histocompatibility antigen D D alpha chain | H2 D1 | 3 | 6 | 13,27 | 4,77E-09 |
| Similar to zeta proteasome chain_ PSMA5 | EG666974 | 3 | 4 | 12,24 | 1,39E-10 |
| Vk protein _Fragment_ |  | 3 | 14 | 9,98 | 1,65E-05 |
| Nucleosome assembly protein 1 like 1 | Nap1l1 | 3 | 6 | 9,94 | 4,28E-06 |
| Dihydrolipoyllysine residue succinyltransferase component of 2_oxoglutarate dehydrogenase complex_ mitochondrial | Dlst | 3 | 6 | 11,12 | 3,37E-06 |
| Proteasome subunit alpha type_3 | Psma3 | 3 | 4 | 7,50 | 3,51E-06 |
| T complex protein 1 subunit epsilon | Cct5 | 3 | 4 | 9,11 | 7,12E-06 |
| Proteasome subunit alpha type_7 | Psma7 | 3 | 6 | 13,23 | 1,54E-06 |
| Inter alpha trypsin inhibitor heavy chain 4 | Itih4 | 3 | 4 | 10,46 | 2,46E-05 |
| Clathrin heavy polypeptide | Cltc | 3 | 4 | 9,41 | 1,94E-06 |
| Complement component 1 q subcomponent C chain | C1qc | 3 | 14 | 12,06 | 9,75E-08 |
| Fetuin_B | Fetub | 3 | 6 | 12,85 | 1,31E-04 |
| Cathepsin B | Ctsb | 3 | 5 | 11,10 | 1,99E-13 |
| Isoform 2 of Sorcin | Sri | 3 | 3 | 7,69 | 1,06E-05 |
| Annexin A1 | Anxa1 | 3 | 3 | 11,27 | 1,05E-05 |
| Complement component 8 alpha polypeptide | C8a | 3 | 5 | 11,91 | 1,90E-09 |
| Serum paraoxonase/arylesterase 1 | Pon1 | 3 | 6 | 12,00 | 2,05E-11 |
| Inter alpha trypsin inhibitor heavy chain H2 | Itih2 | 3 | 5 | 8,68 | 1,71E-05 |
| Inter alpha trypsin inhibitor heavy chain H3 | Itih3 | 3 | 7 | 11,68 | 7,55E-07 |
| Isoform 2 of Collectin 11 | Colec11 | 3 | 4 | 9,88 | 4,44E-05 |
| Moesin | Msn | 3 | 5 | 11,03 | 4,05E-06 |
| Isoform 2 of Leukocyte surface antigen CD47 | Cd47 | 3 | 6 | 12,79 | 4,27E-07 |
| Protein AMBP | Ambp | 3 | 4 | 10,52 | 1,90E-06 |
| Retinol_binding protein 4 | Rbp4 | 3 | 6 | 9,60 | 1,20E-06 |
| Proteasome subunit beta type_5 | Psmb5 | 3 | 5 | 9,14 | 1,33E-04 |
| Proteasome subunit alpha type 2 | Psma2 | 3 | 5 | 10,92 | 2,71E-04 |
| Proteasome subunit beta type 2 | Psmb2 | 3 | 5 | 9,78 | 1,29E-05 |
| Keratin type II cytoskeletal 1 | Krt1 | 3 | 9 | 10,38 | 6,50E-07 |
| Protein kinase C and casein kinase substrate in neurons protein 2 | Pacsin2 | 3 | 7 | 9,98 | 4,98E-04 |
| Transthyretin | Ttr | 3 | 6 | 12,09 | 5,89E-05 |
| RING finger protein 114 | Rnf114 | 3 | 7 | 11,09 | 2,34E-07 |
| Integrin beta 1 | Itgb1 | 3 | 5 | 10,28 | 9,14E-06 |
| Fatty acid synthase | Fasn | 3 | 4 | 9,48 | 4,76E-05 |
| Peroxiredoxin 2 | Prdx2 | 3 | 4 | 9,32 | 1,59E-04 |
| B cell antigen receptor |  | 2 | 10 | 8,01 | 1,50E-05 |
| Isoform Long of Galectin_9 | Lgals9 | 2 | 4 | 7,66 | 4,04E-07 |
| Erythrocyte protein band 4 1 | Epb4 | 2 | 3 | 8,48 | 1,01E-04 |
| L_lactate dehydrogenase | Ldha | 2 | 3 | 6,94 | 3,24E-05 |
| Isoform 2 of 14_3_3 protein theta | Ywhaq | 2 | 3 | 9,41 | 7,26E-10 |
| Ezrin | Ezr | 2 | 3 | 6,21 | 6,70E-05 |
| Ank1 protein | Ank1 | 2 | 4 | 7,47 | 3,35E-06 |
| Ras related protein Rab8B | Rab8b | 2 | 2 | 6,06 | 2,45E-04 |
| Glutamine synthetase | Glul | 2 | 3 | 6,89 | 8,69E-04 |
| Integrin alpha L | Itgal | 2 | 3 | 7,44 | 1,05E-07 |
| Annexin A7 | Anxa7 | 2 | 3 | 8,48 | 6,38E-07 |
| Kininogen 2 isoform 3 | Kng2 | 2 | 4 | 6,52 | 6,46E-05 |
| Apolipoprotein C III | Apoc3 | 2 | 14 | 9,33 | 6,38E-11 |
| Similar to Bridging integrator 2 | Bin2 | 2 | 3 | 6,80 | 1,80E-08 |
| Tubulin beta 5 chain | Tubb5 | 2 | 3 | 6,99 | 5,81E-05 |
| Argininosuccinate lyase | Asl | 2 | 4 | 7,40 | 4,25E-06 |
| Thy1 membrane glycoprotein | Thy1 | 2 | 4 | 6,77 | 9,77E-05 |
| Isoform 2 of Tetraspanin_33 | Tspan33 | 2 | 2 | 6,51 | 6,09E-07 |
| F11 receptor | F11r | 2 | 2 | 5,02 | 9,20E-04 |
| Putative uncharacterized protein | Gnai2 | 2 | 4 | 7,39 | 4,57E-06 |
| Olfactomedin 4 | Olfm4 | 2 | 3 | 8,49 | 7,99E-04 |
| Tubulin alpha 1B chain | Tuba1b | 2 | 4 | 9,61 | 1,02E-07 |
| Adenosylhomocysteinase | Ahcy | 2 | 3 | 5,12 | 5,17E-04 |
| Coagulation factor XII | F12 | 2 | 4 | 6,25 | 1,35E-06 |
| Solute carrier family 1 Neutral amino acid transporter_member 5 | Slc1a5 | 2 | 4 | 5,72 | 1,80E-04 |
| Catalase | Cat | 2 | 4 | 7,31 | 7,47E-08 |
| Proteasome subunit beta type10 | Psmb10 | 2 | 4 | 10,36 | 4,56E-08 |
| Phosphorylase | Pygl | 2 | 2 | 4,90 | 1,74E-05 |
| Polymeric immunoglobulin receptor | Pigr | 2 | 3 | 5,32 | 1,30E-06 |
| Maltase glucoamylase | Mgam | 2 | 3 | 5,77 | 1,21E-05 |
| Forkhead box protein J2 | Foxj2 | 2 | 3 | 5,48 | 2,01E-04 |
| Macrophage colony stimulating factor 1 receptor | Csf1r | 2 | 3 | 7,03 | 5,15E-06 |
| Annexin A2 | Anxa2 | 2 | 3 | 6,25 | 2,97E-04 |
| Alpha enolase | Eno1 | 2 | 2 | 7,29 | 2,48E-05 |
| Peroxiredoxin 1 | Prdx1 | 2 | 2 | 4,12 | 9,14E-04 |
| Annexin A11 | Anxa11 | 2 | 3 | 7,04 | 5,15E-04 |
| Isoform 2 of Complement factor D | Cfd | 2 | 3 | 9,14 | 5,74E-05 |
| Isoform 2 of NSFL1 cofactor p47 | Nsfl1c | 2 | 3 | 7,54 | 2,84E-08 |
| 26S proteasome non ATPase regulatory subunit 2 | Psmd2 | 2 | 4 | 8,64 | 4,65E-05 |
| Beta actin like protein 2 | Actbl2 | 2 | 6 | 7,94 | 4,03E-04 |
| C reactive protein | Crp | 2 | 4 | 8,92 | 4,03E-08 |
| Aquaporin 1 | Aqp1 | 2 | 4 | 11,91 | 1,73E-11 |
| Brain acid soluble protein 1 | Basp1 | 2 | 4 | 8,11 | 6,17E-11 |
| Procollagen lisien 2_oxoglutarate 5_dioxygenase 1 | Plod1 | 2 | 2 | 4,64 | 1,50E-04 |
| Beta_D_galactosidase fusion protein |  | 2 | 29 | 6,04 | 2,94E-04 |
| BTB/POZ domain containing protein KCTD12 | Kctd12 | 2 | 3 | 6,13 | 1,88E-06 |
| Carbohydrate sulfotransferase 1 | Chst1 | 2 | 3 | 7,55 | 5,56E-06 |
| Keratin type II cytoskeletal 8 | Krt8 | 2 | 3 | 4,59 | 1,21E-05 |
| Glutathione S_transferase mu 1 | Gstm1 | 1 | 2 | 3,51 | 4,31E-07 |
| Ubc protein | Uba52 | 1 | 2 | 3,57 | 1,23E-05 |
| Ribosome binding protein 1 isoform a | Rrbp1 | 1 | 2 | 3,52 | 5,29E-06 |
| Syntaxin 4 | Stx4a | 1 | 1 | 3,88 | 3,55E-05 |
| Lymphocyte antigen 6C2 | Ly6c2 | 1 | 1 | 5,39 | 2,99E-08 |
| Keratin type II cytoskeletal 6A | KRT6A | 1 | 2 | 4,00 | 6,81E-04 |
| Haptoglobin | Hp | 1 | 2 | 3,95 | 2,04E-07 |
| Alpha_2_glycoprotein 1 zinc precursor | Azgp1 | 1 | 2 | 4,87 | 5,96E-08 |
| Isoform 2 of Otoferlin | Otof | 1 | 5 | 3,90 | 6,10E-04 |
| Calpain small subunit 1 | Capns1 | 1 | 1 | 3,50 | 1,19E-04 |
| Heat shock protein HSP 90 alpha | Hsp90aa1 | 1 | 2 | 5,17 | 1,84E-13 |
| RAS_related C3 botulinum substrate 3 isoform CRA a | Rac3 | 1 | 1 | 3,51 | 4,31E-05 |
| Plasma membrane calcium ATPase 3 | Atp2b3 | 1 | 2 | 5,17 | 1,75E-09 |
| 40S ribosomal protein S3 | Rps3 | 1 | 1 | 3,83 | 2,18E-09 |
| Proteasome Prosome macropain 26S subunit ATPase 3 | Psmc3 | 1 | 1 | 3,73 | 6,34E-05 |
| Isoform 1 of CD97 antigen | Cd97 | 1 | 2 | 4,18 | 2,08E-07 |
| Angiotensinogen | Agt | 1 | 1 | 4,11 | 3,40E-05 |
| Integrin alpha 2 | Itga2 | 1 | 1 | 3,78 | 3,52E-05 |
| Peptidyl prolyl cis_trans isomerase B | Ppib | 1 | 2 | 3,61 | 1,05E-04 |
| Alpha actinin 4 | Actn4 | 1 | 2 | 4,15 | 6,84E-08 |
| Fermitin family homolog 3 | Fermt3 | 1 | 1 | 4,54 | 1,25E-06 |
| Beta_2_microglobulin | B2m | 1 | 2 | 3,61 | 1,49E-06 |
| 26S protease regulatory subunit 4 | Psmc1 | 1 | 2 | 4,21 | 3,48E-05 |
| Probable G protein coupled receptor 160 | Gpr160 | 1 | 2 | 4,18 | 3,71E-08 |
| 78 kDa glucose regulated protein | Hspa5 | 1 | 2 | 3,53 | 6,98E-04 |
| Vascular non_inflammatory molecule 3 | Vnn3 | 1 | 1 | 3,59 | 8,04E-05 |
| Alpha 1 acid glycoprotein 1 | Orm1 | 1 | 8 | 4,59 | 4,72E-11 |
| Guanine nucleotide binding protein G | Gnas | 1 | 1 | 3,66 | 1,13E-04 |
| Rho related GTP binding protein RhoG | Rhog | 1 | 2 | 4,05 | 2,21E-06 |
| 60S ribosomal protein L32 | Rpl32 | 1 | 2 | 4,61 | 7,58E-09 |
| Alpha amylase 1 | Amy1 | 1 | 3 | 4,72 | 2,08E-08 |
| Ribosomal protein L14 | Rpl14 | 1 | 2 | 3,62 | 1,75E-06 |
| Annexin A5 | Anxa5 | 1 | 2 | 5,44 | 5,81E-08 |
| Isoform 2 of N_acetyltransferase MAK3 homolog | Nat12 | 1 | 4 | 3,66 | 3,23E-04 |
| Dehydrogenase/reductase SDR family member 11 | Dhrs11 | 1 | 1 | 3,71 | 2,27E-06 |
| Isoform 4 of Calcium/calmodulin_dependent protein kinase type II delta chain | Camk2d | 1 | 2 | 4,64 | 8,31E-10 |
| Similar to SIRP beta 1 like 1 protein |  | 1 | 16 | 3,50 | 3,09E-05 |
| Cofilin 1 | Cfl1 | 1 | 2 | 3,80 | 3,84E-08 |
| Heat shock protein HSP 90 beta | Hsp90ab1 | 1 | 1 | 4,94 | 2,17E-11 |
| 26S protease regulatory subunit 8 | Psmc5 | 1 | 1 | 4,00 | 1,62E-07 |
| Granulin | Grn | 1 | 2 | 4,43 | 1,65E-10 |
| Similar to H4a 3 coding region | LOC630322 | 1 | 6 | 4,50 | 9,70E-05 |
| Dynein cytoplasmic 1 intermediate chain 2 | Dync1i2 | 1 | 2 | 3,83 | 1,20E-09 |
| Isoform Long of Tripeptidyl peptidase 2 | Tpp2 | 1 | 4 | 5,00 | 8,51E-05 |
| Tetraspanin 8 isoform CRA a | Tspan8 | 1 | 2 | 3,99 | 2,14E-07 |
| 60S ribosomal protein L18 | Rpl18 | 1 | 2 | 3,97 | 5,34E-07 |
| Large neutral amino acids transporter small subunit 1 | Slc7a5/LAT1 | 1 | 2 | 3,51 | 1,68E-10 |
| Isoform 2 of Reelin | Reln | 1 | 1 | 4,25 | 4,43E-05 |
| Testis expressed gene 15 | Tex15 | 1 | 5 | 4,07 | 1,13E-04 |
| Thioredoxin domain containing protein 5 | Txndc5 | 1 | 2 | 4,66 | 2,28E-08 |
| Adiponectin | Adipoq | 1 | 2 | 3,87 | 4,36E-06 |
| Heparin cofactor 2 | Serpind1 | 1 | 1 | 4,31 | 1,13E-04 |
| Apolipoprotein C_II | Apoc2 | 1 | 1 | 4,03 | 4,99E-08 |
| Arginyl_tRNA synthetase_ cytoplasmic | Rars | 1 | 1 | 3,80 | 2,14E-07 |
| Profilin | Pfn1 | 1 | 1 | 3,74 | 3,14E-05 |
| Triosephosphate isomerase | Tpi1 | 1 | 2 | 3,84 | 9,26E-09 |
| Proto_oncogene tyrosine_protein kinase LCK | Lck | 1 | 1 | 5,27 | 4,72E-11 |
| Early endosome antigen 1 | Eea1 | 1 | 1 | 5,05 | 3,41E-08 |
| Hepatocyte growth factor activator | Hgfac | 1 | 2 | 3,69 | 5,54E-07 |
| Putative deoxyribose_phosphate aldolase | Dera | 1 | 1 | 3,99 | 3,38E-04 |
| BAG family molecular chaperone regulator 2 | Bag2 | 1 | 1 | 4,66 | 7,33E-10 |
| Putative uncharacterized protein | 2410002F23Rik | 1 | 2 | 3,87 | 1,17E-09 |
| Vasodilator_stimulated phosphoprotein | Vasp | 1 | 2 | 3,77 | 7,08E-06 |
| Proteasome subunit beta type 7 | Psmb7 | 1 | 1 | 4,18 | 3,16E-07 |
| Monocarboxylate transporter 1 | Slc16a1 | 1 | 4 | 4,52 | 1,12E-06 |
| Aldehyde dehydrogenase 7 family member A1 isoform a | Aldh7a1 | 1 | 1 | 4,27 | 5,27E-06 |
| Dipeptidyl peptidase 4 | Dpp4 | 1 | 1 | 4,17 | 5,61E-05 |
| Elongation factor 1 beta | Eef1b2 | 1 | 2 | 3,80 | 1,84E-07 |
| Myeloid_associated differentiation marker | Myadm | 1 | 2 | 4,72 | 1,21E-07 |

* Xcorr sum – Sum of the cross-correlation scores of individual peptides from each identified protein.
